# Voltage-controlled interlayer coupling in perpendicularly magnetized magnetic tunnel junctions

**DOI:** 10.1038/ncomms15232

**Published:** 2017-05-16

**Authors:** T. Newhouse-Illige, Yaohua Liu, M. Xu, D. Reifsnyder Hickey, A. Kundu, H. Almasi, Chong Bi, X. Wang, J. W. Freeland, D. J. Keavney, C. J. Sun, Y. H. Xu, M. Rosales, X. M. Cheng, Shufeng Zhang, K. A. Mkhoyan, W. G. Wang

**Affiliations:** 1 Department of Physics, University of Arizona, Tucson, Arizona 85721, USA; 2 Quantum Condensed Matter Division, Oak Ridge National Laboratory, Oak Ridge, Tennessee 37831, USA; 3 Department of Chemical Engineering and Materials Science, University of Minnesota, Minneapolis, Minnesota 55455, USA; 4 Department of Physics, Bryn Mawr College, Bryn Mawr, Pennsylvania 19010, USA; 5 Advanced Photon Source, Argonne National Laboratory, Argonne, Illinois 60439, USA

## Abstract

Magnetic interlayer coupling is one of the central phenomena in spintronics. It has been predicted that the sign of interlayer coupling can be manipulated by electric fields, instead of electric currents, thereby offering a promising low energy magnetization switching mechanism. Here we present the experimental demonstration of voltage-controlled interlayer coupling in a new perpendicular magnetic tunnel junction system with a GdO_*x*_ tunnel barrier, where a large perpendicular magnetic anisotropy and a sizable tunnelling magnetoresistance have been achieved at room temperature. Owing to the interfacial nature of the magnetism, the ability to move oxygen vacancies within the barrier, and a large proximity-induced magnetization of GdO_*x*_, both the magnitude and the sign of the interlayer coupling in these junctions can be directly controlled by voltage. These results pave a new path towards achieving energy-efficient magnetization switching by controlling interlayer coupling.

## Abstract (web summary)

Exploring electric field controlled magnetism enables high efficiency and low energy consumption spintronic devices. Here, by manipulating oxygen vacancies and magnetic moment, the authors achieve voltage control of magnetic interlayer coupling in GdO_*x*_ based magnetic tunnel junctions.

In spintronics^1^,^2^, information is stored with the spin orientations of nanomagnets. One focus of current spintronics research is lowering the switching energy of nanomagnets in magnetic tunnel junctions (MTJs)^3^,^4^,^5^,^6^,^7^. A nanomagnet can be switched by a magnetic field governed by Ampere's law, or by current-induced spin transfer torques^8^,^9^ and spin–orbit torques^10^,^11^. For future spintronic applications, it is highly desirable to accomplish magnetization switching with voltage^12^, which, by eliminating Joule heating, could markedly reduce the switching energy. Indeed, in MTJs with perpendicular easy axes (pMTJs), a switching energy that is more than 10 times smaller than that of spin transfer torques has been demonstrated with the voltage-controlled magnetic anisotropy (VCMA) effect^13^,^14^,^15^,^16^,^17^. Owing to the precessional nature of VCMA switching, however, the duration of the voltage pulses and the size distribution of the nanomagnets have to be precisely controlled, which is a disadvantage for practical applications^18^. Other voltage-based switching scenarios involving multiferroic or magnetoelectric materials also have been intensively investigated^12^,^19^,^20^,^21^,^22^, although the incorporation of these materials into an MTJ structure working at room temperature (RT) has yet to be demonstrated.

In addition to VCMA, another possible way to achieve voltage-induced switching in pMTJs at RT is by controlling the interlayer coupling between the hard and soft ferromagnetic (FM) layers, as schematically illustrated in Fig. 1. Interlayer exchange coupling is one of the most central phenomena in spintronics. The observation of antiferromagnetically (AFM) coupled FM layers through nonmagnetic (NM) spacers^23^ and the subsequent discovery of giant magnetoresistance^24^ led to the birth of spintronics. It has been proposed that by controlling the reflection coefficients of spin-up and spin-down electrons at the interfaces^25^,^26^ or by modifying the induced charge and magnetization in FM/NM/FM multilayers^27^,^28^, the interlayer coupling and thus the magnetoresistance can be changed with applied voltage. To date, however, these predictions have not been experimentally realized.

Here we report the experimental demonstration of voltage-controlled interlayer coupling (VCIC) in an MTJ structure. This unprecedented control of interlayer coupling is realized in a new pMTJ system with a unique rare earth oxide, GdO_*x*_, tunnel barrier. A sizable RT tunnelling magnetoresistance (TMR) as well as a large perpendicular magnetic anisotropy (PMA) was simultaneously obtained in these MTJs. Owing to the interfacial nature of the PMA, we show that the magnetic properties of the MTJs can be manipulated by voltage, similarly to CoFeB/MgO/CoFeB junctions^14^. More importantly, thanks to the ability to move oxygen vacancies within the GdO_*x*_ tunnel barrier and a surprisingly large induced net magnetic moment of the Gd ions, the interlayer coupling in this system can be reversibly and deterministically switched between AFM and FM states by applied voltage.

## Results

### Structure and basic magnetic properties

The structure of the pMTJs is Si/SiO_2_/Ta(8 nm)/Ru(10 nm)/Ta(7 nm)/Co_20_Fe_60_B_20_(0.7–0.9 nm)/GdO_*x*_(1–3.5 nm)/Co_20_Fe_60_B_20_(1.5–1.6 nm)/Ta(7 nm)/Ru(20 nm). The films were deposited in a 12-source ultra-high vacuum magnetron sputtering system with a base pressure of 10^−9^ Torr. After deposition of the multilayers, MTJs in circular shapes with diameters (*D*) ranging from 3 to 20 μm were fabricated and measured in a four-wire geometry. A positive applied voltage in this work corresponds to electrons tunnelling from the bottom FM to the top FM.

The microstructure of the GdO_*x*_-pMTJs was investigated by transmission electron microscopy (TEM) as shown in Fig. 2a. Smooth interfaces between the FM electrodes and the tunnel barrier can be seen, similar to MTJs with AlO_*x*_ (refs 3, 4) and MgO (refs 5, 6) barriers. The oxide layer shows no crystalline texture, indicating that the barrier is amorphous, as with most MTJs with AlO_*x*_ barriers^3^,^4^. X-ray diffraction on thick gadolinium oxide layers shows that it forms the cubic Gd_2_O_3_ phase^29^. It is difficult to determine, however, the exact oxidation state of the Gd in the amorphous barrier. Thus, the tunnel barrier in this study is referred to as GdO_*x*_. More details of the sample characterization is shown in Supplementary Fig. 1.

A representative TMR curve from these GdO_*x*_-pMTJs is shown in Fig. 2b. The core structure of this MTJ is CoFeB(0.85 nm)/GdO_*x*_(2.1 nm)/CoFeB(1.6 nm) with *D*=7 μm. It shows very sharp resistance switching and a flat antiparallel state, characteristic of MTJs with perpendicular easy axes. A sizable RT TMR of 15% has been observed in this MTJ, where TMR=(*R*_AP_−*R*_P_)/*R*_P_, *R*_AP_ and *R*_P_ being the resistance in the antiparallel and parallel configurations, respectively. Previously, magnetoresistance in MTJs with a GdO_*x*_ barrier was only achieved at liquid helium temperature or below, using thick FM electrodes with in-plane anisotropy^30^,^31^. Notably, the lattice constant of cubic Gd_2_O_3_ has only a 5% mismatch with that of CoFe. Therefore, as in MTJs with both MgO(001) (refs 5, 6) and Al_2_O_3_(001) (ref. 32) barriers where a TMR above 200% has been achieved, a substantially large TMR potentially could be obtained with an epitaxial Gd_2_O_3_ barrier, for example, in CoFe(001)/Gd_2_O_3_(001) MTJs. The saturation magnetization and anisotropy field of the Ta/CoFeB/GdO_*x*_ structure have been determined to be 800 e.m.u. cm^−3^ and 6,000 Oe, respectively (Supplementary Note 1). These values give rise to a PMA energy density of 2.6 × 10^6^ erg cm^−3^, which is comparable with CoFeB/MgO-pMTJs^7^,^33^. Owing to the interfacial nature of the PMA, the coercivity (*H*_C_) of the CoFeB layers can be directly manipulated by voltage. As shown in Supplementary Note 2, a positive voltage of 0.7 V reduces the *H*_C_ of the top CoFeB layer and enhances the *H*_C_ of the bottom CoFeB layer, as compared to the TMR curve measured at 10 mV. (The top CoFeB has smaller switching fields, as demonstrated in Supplementary Fig. 3.) This is the VCMA effect as previously observed in CoFeB/MgO-pMTJs^14^,^15^, which is attributed to the electric field-induced redistribution of the electron densities among different *d* orbitals in the FMs. These results establish CoFeB/GdO_*x*_ as the only MTJ system other than CoFeB/MgO that exhibits RT TMR, PMA and VCMA effects to date.

### Voltage-controlled interlayer coupling

Owing to the large ionic mobility of oxygen vacancies in GdO_*x*_, it has been shown that both the magnetic anisotropy field (*H*_A_) and saturation magnetization (*M*_S_) in Pt/Co/GdO_*x*_ multilayers can be manipulated by controlling the oxidation state of the Co through external electric fields in a nonvolatile manner^29^,^34^,^35^. As a result, the Co can be reversibly changed from an optimally oxidized state with a strong PMA to a metallic state with an in-plane magnetic anisotropy or to a fully oxidized state with nearly zero magnetization, depending on the polarity of the applied voltage. While the change of *M*_S_ and *H*_A_ can be probed by anomalous Hall effect^29^ or Kerr rotation^34^ measurements, it is generally understood to be much more challenging to realize such magneto-ionic control of magnetism in an MTJ structure because TMR has more stringent requirements on the interface quality and tunnelling spin polarization. Here we demonstrate such an effect in pMTJs. The apparent coercivities of the top and bottom CoFeB layers, measured in full TMR loops, are defined as *H*_C-TOP_ and *H*_C-BOT_. These coercivities are dependent on the interlayer coupling, as opposed to the intrinsic coercivities 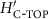 and 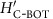, which are not influenced by the interlayer coupling. The initial full TMR curve (black) of a GdO_*x*_-pMTJ is shown in Fig. 3a. After application of a +0.5 V setting voltage (*V*_SET_) for 300 s at an elevated temperature of 150 °C, the TMR was measured when the junction was returned to RT. As a result of the magneto-ionic effect, both *H*_C-TOP_ and *H*_C-BOT_ are reduced, and the MTJ shows a narrow antiparallel plateau. Subsequently, the *H*_C-TOP_ and *H*_C-BOT_ can be restored to almost their original values by applying *V*_SET_=−0.5 V for 25 s at 150 °C. Note that the TMR curves were measured at RT under a voltage of <10 mV after the removal of *V*_SET_. Therefore, this effect is distinctly different from the VCMA effect shown in Supplementary Note 2, where the change of *H*_C_ is only observed during the application of large voltages. Realization of this nonvolatile (Supplementary Fig. 5) control of TMR immediately opens up new applications such as microwave devices and magnetic field sensors with variable sensitivity and range.

**Figure 1:**
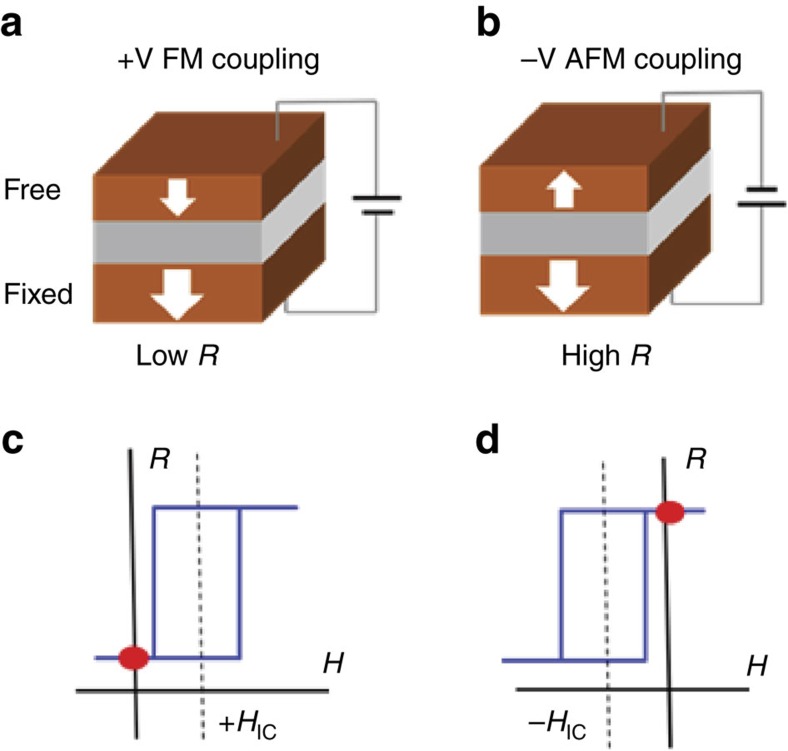
Schematic switching of an MTJ by VCIC. (**a**,**b**) The core structure of an MTJ consists of a fixed FM layer and a free FM layer separated by a tunnel barrier. The MTJ can be set to a low (high)-resistance state with FM (AFM) coupling by applying a positive (negative) voltage pulse under zero magnetic field. (**c**,**d**) The FM (AFM) coupled state can be revealed by the positive (negative) shift of the minor TMR loop, measured with the bottom FM kept pointing down while changing the spin orientation of the top FM by sweeping an external magnetic field. The two resistance values at zero magnetic field are marked by red dots, which correspond to the two states in the upper panels.

**Figure 2:**
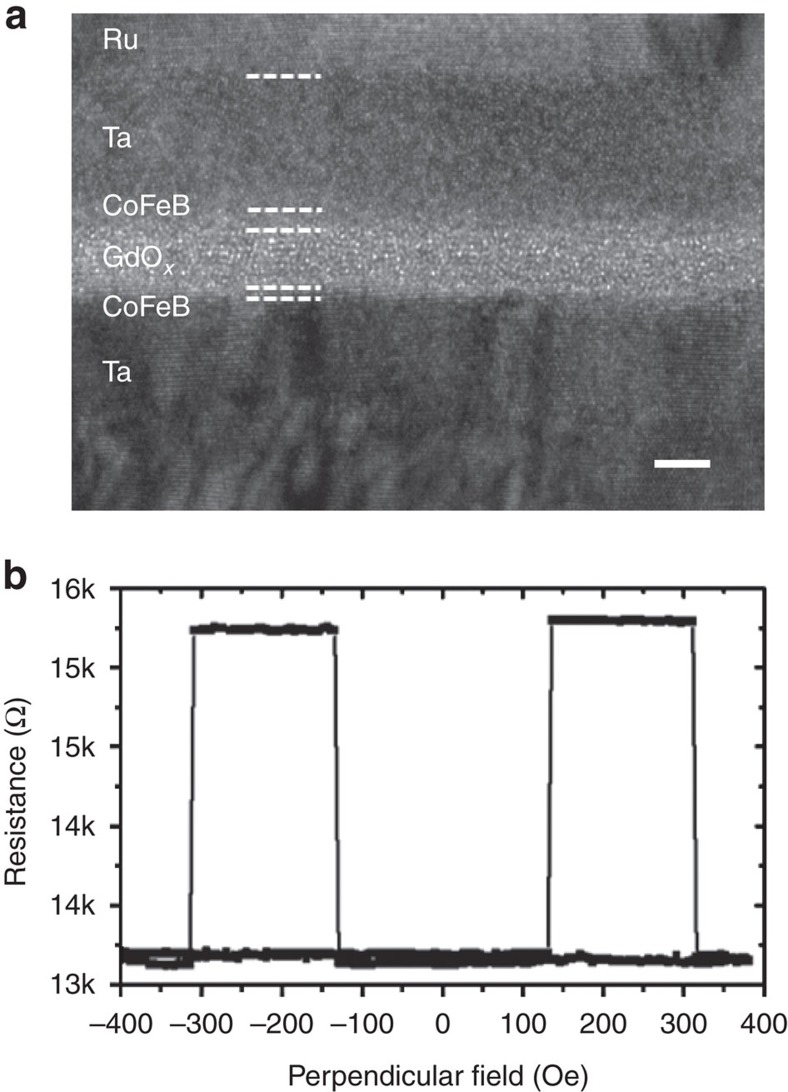
Microstructure and TMR curve of a GdO_*x*_-pMTJ. (**a**) High-resolution cross-sectional TEM image of an MTJ. Both the GdO_*x*_ barrier and CoFeB electrodes are amorphous in the present samples. Scale bar is 3 nm in length. (**b**) A representative RT magnetoresistance curve of a GdO_*x*_-pMTJ showing a TMR of 15%.

**Figure 3:**
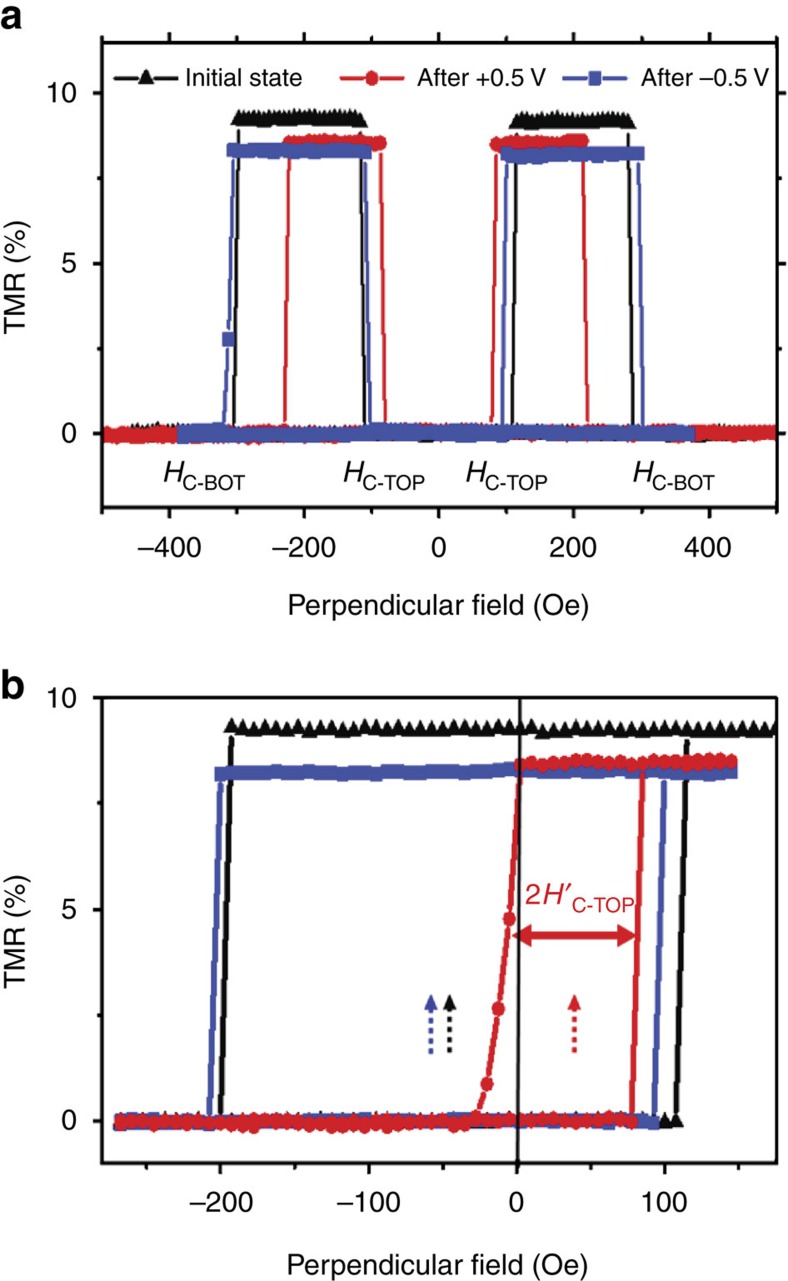
VCIC in a GdO_*x*_-pMTJ. (**a**) The initial full TMR curve (black), and after the application of *V*_SET_=0.5 V for 300 s at 150 °C (red) and subsequently *V*_SET_=−0.5 V for 25 s (blue) at 150 °C. The core structure of the pMTJ is CoFeB(0.85 nm)/GdO_*x*_(3.1 nm)/CoFeB(1.6 nm). All TMR curves were measured at RT under a low bias of 5–10 mV. The apparent coercivities of the top and bottom CoFeB layers are labelled as *H*_C-TOP_ and *H*_C-BOT_. (**b**) Corresponding minor TMR curves for the three states showing *H*_IC_ can be changed from −42 Oe in the initial state to +37 Oe by +*V*_SET_ and subsequently changed to −51 Oe by −*V*_SET_. The dashed arrows indicate the position of *H*_IC_ in the three states. The intrinsic coercivity of the top CoFeB is labelled as 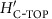. The minor loops were measured by switching only the magnetization of the top CoFeB layer while keeping the magnetization of the bottom CoFeB pointing down.

Next, the minor TMR loops for the three states are shown in Fig. 3b. The interlayer coupling fields (*H*_IC_) are revealed by the centre position of the loops (marked by arrows in Fig. 3b). The initial state of the interlayer coupling is negative (AFM), with *H*_IC_=−42 Oe. After the application of +*V*_SET_, two striking features emerge. First, 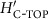 is markedly reduced (from 150 to 45 Oe), much more pronounced than the reduction of *H*_C-TOP_ (from 100 to 80 Oe). Second and most surprisingly, the sign of the interlayer coupling is changed; namely, the coupling is now positive (FM) with *H*_IC_=+37 Oe. Remarkably, with the application of −*V*_SET_, the centre of the minor loop can be shifted back to the left, with an even more negative *H*_IC_ of −51 Oe. Results in Fig. 3b represent the first demonstration of VCIC in an MTJ system, where both the magnitude and sign of the interlayer coupling can be changed by applied voltage. The fact that *H*_C-TOP_ is much larger (smaller) than 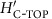 in the FM (AFM) coupling configuration is in full agreement with VCIC, since *H*_C_=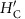
+*H*_IC_. *H*_C_ is larger (smaller) than 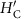 when the sign of *H*_IC_ is set by voltage to be positive (negative). Therefore, the change of *H*_C-TOP_ in Fig. 3a is much smaller compared to the change of 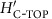 in Fig. 3b. This remarkable observation of VCIC is related to the unique properties of the GdO_*x*_ barrier. For comparison, we performed the voltage experiments with MgO-pMTJs under the same conditions. As expected, no change of *H*_IC_ was observed for MgO-pMTJs, as shown in Supplementary Note 3. One may also note the resemblance between the VCIC effect shown in Figs 1 and 3b with the voltage-controlled exchange bias effect observed in Cr_2_O_3_/(Pd/Co)_*X*_ structures^19^. The possibility of our results being due to voltage-controlled exchange bias has been discounted based on the symmetric shape of the full loops both before and after application of *V*_SET_, as shown in Fig. 3a. Exchange bias adds asymmetry to the full TMR loop not just the minor loop in contrast to what is seen with our samples.

**Figure 4:**
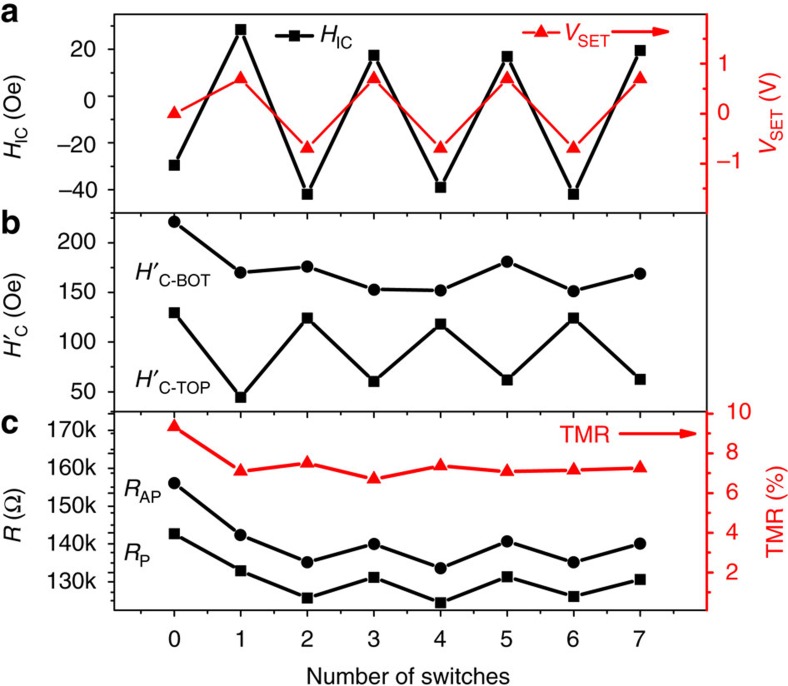
Reversible and deterministic control of interlayer coupling by voltage. (**a**) Continuous switching of *H*_IC_ by *V*_SET_. (**b**) Corresponding intrinsic switching fields of the top and bottom CoFeB electrodes. (**c**) Corresponding change in TMR, *R*_AP_, and *R*_P_ during the switching process.

This control of interlayer coupling is fully reversible and deterministic as further demonstrated in Fig. 4a; namely, FM coupling can always be achieved by +*V*_SET_ and AFM coupling by −*V*_SET_. The corresponding intrinsic switching fields 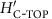 and 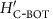 are presented in Fig. 4b. The structure of this sample is CoFeB(0.8 nm)/GdO_*x*_(3 nm)/CoFeB(1.5 nm). Clearly, the value of 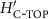 is always decreased (increased) by applying a positive (negative) voltage. This is consistent with the picture of voltage-driven oxidation where the top CoFeB is increasingly oxidized (reduced) by oxygen ions due to the positive (negative) electric field applied to the GdO_*x*_ barrier. Ideally, 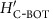 should behave opposite to 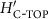, which indeed was the case after the fourth switching, although the change is smaller than that of 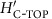. The non-ideal behaviour of 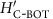 in the beginning and its smaller change in magnitude are likely related to the weaker effect that voltage has on the bottom CoFeB layer, due to its much stronger PMA as a result of our fabrication method. It can now be understood that the AFM coupling state corresponds to a large antiparallel plateau (larger difference between *H*_C-TOP_ and *H*_C-BOT_) and that the FM coupling state corresponds to a smaller antiparallel plateau (smaller difference between *H*_C-TOP_ and *H*_C-BOT_) in the TMR curves, as shown in Fig. 3a, further validating the control of interlayer coupling by voltage. Finally, both *R*_P_ and *R*_AP_ can be systematically controlled by the applied voltages, while the TMR stayed nearly flat despite the change in sign of the interlayer coupling. Although in this proof-of-concept study, *V*_SET_ was applied at 150–180 °C for at least a few tens of seconds, the speed of this effect can, in principle, be markedly improved upon optimization. For example, it has been demonstrated that resistance changes due to voltage-driven O^2−^ motion can be very fast (ns) in memristors^36^.

The early studies on interlayer coupling were focused on metallic spin valves^23^,^24^ with interest later shifting to MTJs^37^,^38^,^39^,^40^,^41^ due to the larger ratio of TMR compared to that of giant magnetoresistance. The coupling was described by the quantum interferences of wave functions due to spin-dependent reflections at the FM/NM interfaces^42^ or by the torques exerted by spin-polarized conduction electrons^43^. AFM coupling in MTJs in most cases is related to oxygen vacancies^38^,^44^ and is also fundamentally more interesting because FM coupling could simply result from pinholes in the tunnel barrier or orange peel effects. For example, in high-quality epitaxial Fe/MgO/Fe MTJs, the observed negative *H*_IC_ was explained by interlayer coupling mediated through oxygen vacancies in the MgO barrier^38^. It was further demonstrated in a density functional theory calculation that not only the magnitude but also the sign of the interlayer coupling could be changed by controlling the distribution of oxygen vacancies in the MgO barrier^44^. However, these studies were carried out on MTJ systems with in-plane magnetic anisotropy, which cannot be directly applied to our pMTJs, where both the *H*_A_ and *M*_S_ of the FMs can be directly modified through VCMA or magneto-ionic effects. The pioneering study on AFM coupling in pMTJs was carried out on a Co/MgO/Co system^39^, where an oscillatory *H*_IC_ was observed with varying Co thickness and explained by Fabry–Perot interferences within Bruno's theory^40^. A model based on roughness-induced orange peel coupling was successfully used to explain the interlayer coupling in (Co/Pt)-Ru-(Co/Pt) spin valves with PMA^45^. According to this model, the AFM coupling is enhanced when the PMA of the FMs is increased. In our GdO_*x*_-pMTJs, however, a decrease in AFM coupling was observed when the PMA of the CoFeB layers was increased (Supplementary Note 4). Therefore, the interlayer coupling in our pMTJs cannot be explained by the model based on roughness-induced Neel-type coupling.

### *In situ* X-ray magnetic circular dichroism study

**Figure 5:**
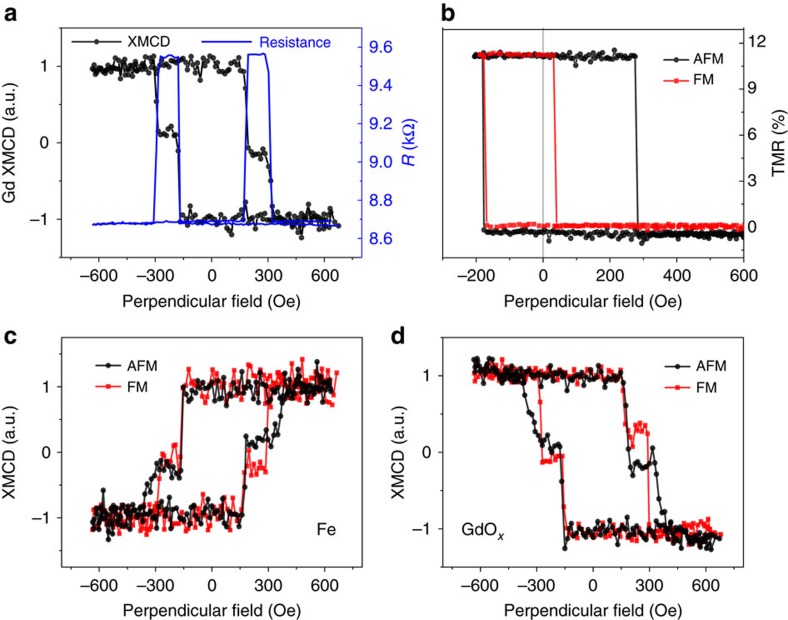
Simultaneous measurement of TMR and XMCD in a GdO_*x*_-pMTJ. (**a**) The magnetic field dependence of junction resistance and the XMCD signal measured at the Gd M_5_-edge (1,184.7 eV). (**b**) Minor TMR loops of the pMTJ set in the AFM and FM coupling states. (**c**) XMCD hysteresis loops measured at the Fe L_3_-edge (707.2 eV) for the AFM and FM states in **b**. (**d**) XMCD hysteresis loops measured at the Gd M_5_-edge (1,184.7 eV) for the AFM and FM states in **b**.

To shed more light on the observed VCIC, we designed an *in situ* experiment where TMR, X-ray absorption spectroscopy (XAS) and X-ray magnetic circular dichroism (XMCD) were measured simultaneously at beam line 4-ID-C of the Advanced Photon Source. Details of the XAS/XMCD experiment are presented in Supplementary Note 5. The pMTJ studied has a 3.3 nm GdO_*x*_ barrier, with a parallel resistance of 8.7 kΩ and a TMR of 11% as plotted in Fig. 5a. Clear XAS and XMCD spectra for the Fe and Co L_2,3_ edges have been observed at photon energies of 700–730 and 770–810 eV, respectively. Surprisingly, a clear XMCD signal was observed at the Gd M_5_ edge (1,180–1,190 eV) under zero magnetic field with an average magnetic moment of 0.6 μ_B_ per Gd, indicating the existence of FM order in the GdO_*x*_ (Supplementary Note 5). Since Gd_2_O_3_ is a weak antiferromagnet with a Neel temperature of <5 K (ref. 46), no ferromagnetism is expected in GdO_*x*_ at RT. Indeed this net XMCD signal becomes vanishingly small in standalone GdO_*x*_ barriers without CoFeB layers, as shown in Supplementary Note 5. In addition, the resistance of the GdO_*x*_-pMTJs exponentially increases with barrier thickness similar to what is seen in high-quality MgO-pMTJs with TMR larger than 160% (ref. 33), and *IV* curve fittings of the MTJs give rise to an average barrier height of about 1 eV in both the FM and AFM states, similar to AlO_*x*_-based junctions (Supplementary Fig. 11). These facts further rule out the existence of any free Gd in the tunnel barrier, although determining the exact oxidation state of the Gd ions in the barrier stays challenging due to its amorphous nature. More strikingly, the magnetic field dependence of the GdO_*x*_ XMCD signal showed two distinct transitions, at fields corresponding to the *H*_C-TOP_ and *H*_C-BOT_ of the TMR curve, as shown in Fig. 5a. This unexpected observation of FM GdO_*x*_ suggests that the magnetic signal is induced by the CoFeB layers through the proximity effect as observed in Pt/Fe (ref. 47). The appearance of two distinct jumps in the XMCD hysteresis loop arising from the single GdO_*x*_ barrier layer supports this notion, reflecting the short-range nature of proximity coupling. Furthermore, the direction of the induced FM moment of GdO_*x*_ is antiparallel to that of Fe and Co (Supplementary Note 5). This AFM locking of moments between Fe and Gd ions explains why the induced moments of GdO_*x*_ switch at the exact same magnetic fields as CoFeB (shown in Fig. 5a). This large induced magnetization of the GdO_*x*_ barrier is probably related to the giant spin moment of Gd^3+^ (*S*=7/2), which has exhibited exotic behaviours such as generating a Zeeman splitting field of 4 T even when only 1.5 T was applied to Gd_2_O_3_/Al/Al_2_O_3_/Fe junctions^48^.

Now we can take a closer look at the VCIC effect. The pMTJ was set to AFM and FM coupled states as shown in the minor TMR loops in Fig. 5b. XMCD full hysteresis loops for Fe and Gd ions are displayed in Fig. 5c,d. First, the Gd loops in the AFM and FM states generally resemble the shape of the Fe loops but with opposite sign, as anticipated from the AFM coupling between the Gd ions and the CoFeB. Second, the hysteresis loops for both Fe and Gd in the AFM state exhibit a wider plateau between the two transitions, which corresponds to the high-resistance state of the pMTJ, agreeing with what is presented in Fig. 3a. Third and most significantly, these measurements directly confirm our hypothesis of voltage-driven oxidation/reduction in the GdO_*x*_-pMTJs and the proximity-induced moment of the Gd ions by CoFeB. The transition at 150 Oe in the hysteresis loops of Fig. 5c corresponds to the top CoFeB layer. The relative magnitude of this transition is smaller (larger) after the pMTJ is set to the FM (AFM) state by applying a positive (negative) voltage, exactly as anticipated since a positive (negative) *V*_SET_ drives O^2−^ towards (away from) the top CoFeB layer. Moreover, the magnitudes of the two transitions of the Gd ions in Fig. 5d are proportional to those of the Fe in Fig. 5c, showing a one-to-one correspondence between the moment of GdO_*x*_ and the moment of Fe, which further demonstrates that the observed XMCD signal from the Gd ions is induced by the proximity effect from Fe.

### Proposed phenomenological model

Finally, we attempt to qualitatively explain the observed VCIC in a phenomenological model. The large induced moment of 0.6 μ_B_ per Gd ion, nearly 20 times stronger than the induced moment of Pt in Pt/Fe bilayers^47^, is very interesting. Usually magnetic ions in the barrier will cause spin-flip scattering and are therefore detrimental to TMR. To our benefit, the moments of the Gd ions come from the deeply buried *f* orbitals that have little influence on conduction electrons. They may, however, play an important role in the magnetic properties, for example, the coupling of the two CoFeB layers in a GdO_*x*_-pMTJ. By considering the voltage-driven oxidation level changes of Fe, the large induced moment of the Gd ions that is proportional to the amount of free Fe, and a voltage-dependent distribution of correlated moments in the Gd ions, the VCIC effect can be realized as shown in the model presented in Supplementary Note 6. The *H*_IC_ can be changed between ±100 Oe with appropriate approximations, which is comparable with what has been observed experimentally.

In summary, we presented the first experimental demonstration of VCIC in a new pMTJ system with a GdO_*x*_ barrier. Through the unique combination of strong interfacial PMA, efficient manipulation of oxygen vacancies and a large induced net moment of GdO_*x*_, not only the magnitude but also the sign of the interlayer coupling can be effectively manipulated by applied voltage. These results pave the way towards a new class of spintronic devices where magnetization switching can be accomplished by VCIC.

## Methods

### Sample fabrication

The CoFeB/GdO_*x*_/CoFeB MTJs were deposited on thermally oxidized silicon substrates by a magnetron sputtering system (AJA international) with a base pressure in the range of 10^−9^ Torr. The structure of the samples is Si/SiO_2_/Ta(8 nm)/Ru(10 nm)/Ta(7 nm)/Co_20_Fe_60_B_20_(0.7–0.9 nm)/GdO_*x*_(1–3.5 nm)/Co_20_Fe_60_B_20_(1.5–1.6 nm)/Ta(7 nm)/Ru(20 nm). The thickness of the layers was calibrated by X-ray reflectivity with an uncertainty of 10%. All metallic layers were deposited by d.c. sputtering under Ar pressures of 2–2.5 mTorr. The GdO_*x*_ barrier was deposited by Ar/O_2_ reactive sputtering from a metallic Gd target with an O_2_ partial pressure of 0.19 mTorr. Samples were then patterned using standard photolithography and ion beam etching^49^. The final MTJ pillars had circular shapes with diameters between 3 and 20 μm. The fully patterned MTJs were then annealed for 1–10 min at 250–300 °C in a rapid thermal annealing system. All TMR in this study was measured at RT unless otherwise specified.

To confirm that the observed large effect is related to GdO_*x*_, we investigated the behaviour of a perpendicular MgO MTJ under the same testing conditions. The blanket films were fabricated in the same deposition system with structure of Si wafer/SiO_2_/Ta(8 nm)/Ru(10 nm)/ Ta(7 nm)/Co_20_Fe_60_B_20_(0.86 nm)/MgO(1 nm)/Co_20_Fe_60_B_20_(1.5 nm)/Ta(7 nm)/Ru(20 nm). The films were subsequently patterned into circular MTJs in the same manner as the GdO_*x*_-MTJs. The MgO-MTJs were annealed at 300 °C for 10 min before TMR testing.

### Electron microscopy

The microstructure of the MTJ was imaged using two transmission electron microscopes: an FEI Tecnai G2 F30 at 300 kV for high-resolution conventional TEM and an aberration-corrected (CEOS DCOR probe corrector) FEI Titan G2 60–300 S/TEM equipped with a Schottky X-FEG gun at 200 kV for high-angle annular dark-field scanning TEM. The structural integrity of the MTJ layers after thermal annealing was confirmed with elemental mapping by energy-dispersive X-ray spectroscopy (EDX) using a Super-X quad-SDD windowless in-polepiece EDX detector in the Titan. The probe convergence angle used for scanning TEM imaging and EDX mapping was 21 mrad. Cross-sectional samples were prepared for TEM analysis by milling with a Ga^+^ focused ion beam at 30 and 10 kV in an FEI Quanta 200 three-dimensional dual-beam focused ion beam/scanning electron microscope.

### Data availability

The data supporting these findings are available from the corresponding author on request.

## Additional information

**How to cite this article:** Newhouse-Illige, T. *et al*. Voltage-controlled interlayer coupling in perpendicularly magnetized magnetic tunnel junctions. *Nat. Commun.* **8,** 15232 doi: 10.1038/ncomms15232 (2017).

**Publisher's note**: Springer Nature remains neutral with regard to jurisdictional claims in published maps and institutional affiliations.

## Supplementary Material

Supplementary Information Supplementary Figures, Supplementary Notes and Supplementary References
