# Supplementary material for: Voltage-controlled interlayer coupling in perpendicularly magnetized magnetic tunnel junctions
Source: Nat Commun. 2017 May 16;8:15232. doi: 10.1038/ncomms15232 (PMC5440805; doi:10.1038/ncomms15232)
Supplement: Supplementary Information — Supplementary Figures, Supplementary Notes and Supplementary References [file ncomms15232-s1.pdf]

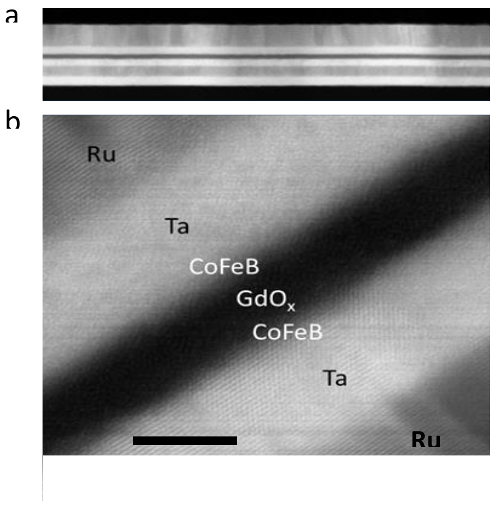

Supplementary Figure 1. HAADF-STEM images of the GdO_x/CoFeB pMTJ film. (a) High-angle annular dark-field scanning transmission electron microscopy (HAADF-STEM) image of a CoFeB/GdO_x/CoFeB pMTJ. The high uniformity of the multilayer structure over a large scale region is visible. **(b)** Core structure of the pMTJ with larger magnification. The scale bar is 5 nm in length The Ru buffer and capping layer exhibit clear layer-by-layer crystalline structure. The bottom Ta layer shows a mixed crystalline and amorphous structure. No clear crystalline grains can be observed in the top Ta layer. Under the present annealing conditions, the amorphous CoFeB are not likely to be crystallized, based on a previous study of MgO-pMTJs[1]. The contrast in HAADF-STEM images is related to atomic number. The amorphous nature of CoFeB and Ta and the intermediate atomic numbers of CoFeB compared to Ta and GdO_x make it hard to clearly distinguish the interface boundaries in the HAADF images. Nevertheless, it can be seen that no crystalline structure exists in the GdO_x barrier, agreeing with the high-resolution conventional transmission electron microscopy image shown in Fig. 2.

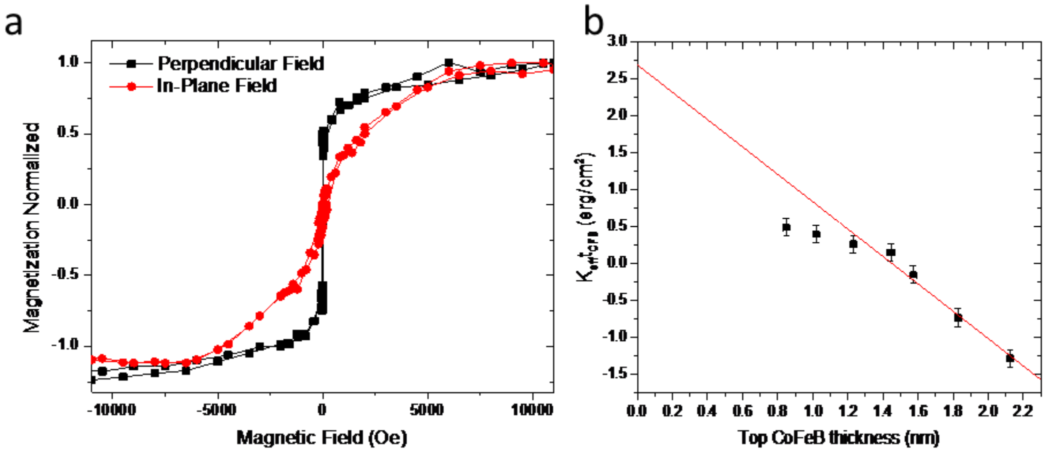

Supplementary Figure 2. Magnetometry curves. (a) Hysteresis loops of the bottom electrode of a pMTJ under in-plane and perpendicular fields. **(b)** Perpendicular anisotropy energy for different thickness of CoFeB. The red line is the linear fit to the data. Error bars are calculated based on combined error in CoFeB thicknesses and anisotropy fields.

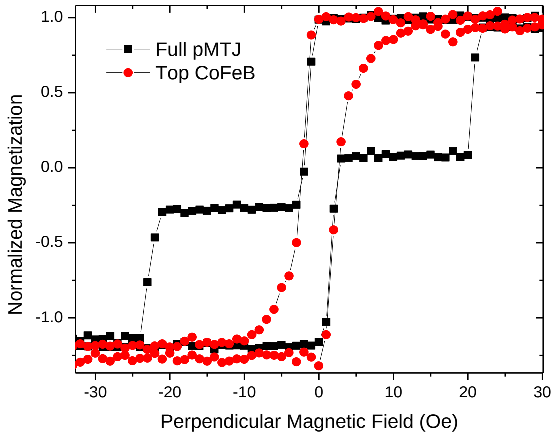

Supplementary Figure 3. Easy axis Magnetometry. Easy axis Hysteresis loops for the full pMTJ structure (black) and the top CoFeB only (red).

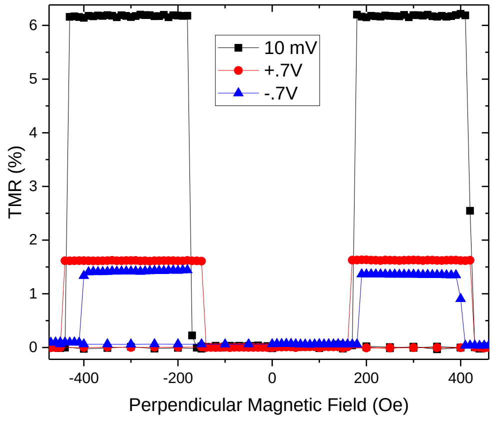

Supplementary Figure 4. VCMA Effect. *TMR* loops of a GdO_x-MTJ showing the VCMA effect. (Black) Low bias state, (Red) Large positive bias enlarges the antiparallel plateau, (Blue) Large negative bias shrinks the antiparallel plateau. The GdO_x thickness of this sample is 2.6 nm.

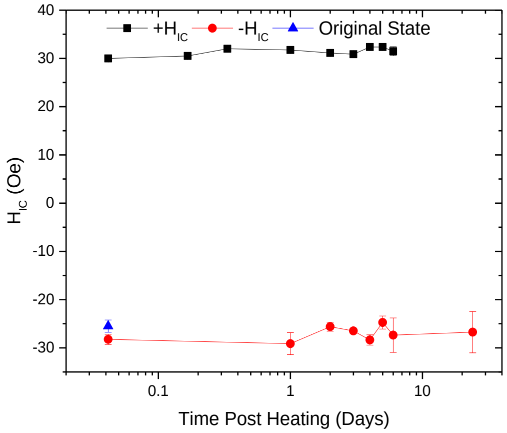

Supplementary Figure 5. Retention of VCIC. H_{IC} as a function of time since last state change. Black is after $V_{set} > 0$, setting the system in a FM state after the initial AFM state (Blue), Red after $V_{set} < 0$, resetting the system to the initial AFM state. The change in $\mathbf{H}_{IC}$ state between AFM and FM is nonvolatile. We use elevated temperature and high bias voltage to change the state but

the state is then measured at RT and low bias. To test the retention of this effect we have measured the $\mathbf{H}_{IC}$ as a function of time after the most recent state change. The $\mathbf{H}_{IC}$ is maintained for at least 3 weeks (as long as the test was run for). Error bars are the standard deviation of 3 repeated curves at each data point. This retention test was carried out on a sample with 2.5 nm GdO_x.

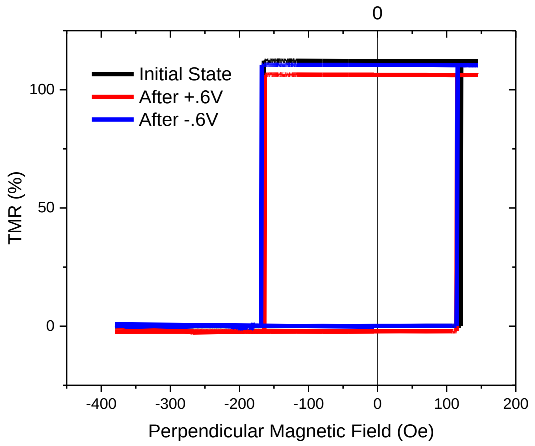

Supplementary Figure 6. Control experiment with MgO-pMTJ. Minor *TMR* curves of an MgO-pMTJ measured at RT in the fresh state (black) and after $+V_{set}$ (red) and $-V_{set}$ (blue).

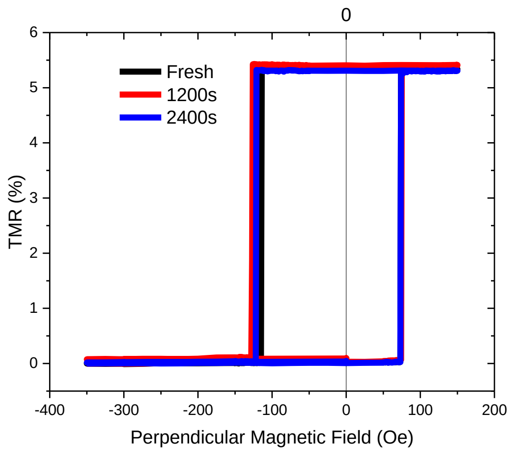

Supplementary Figure 7. Annealing only experiment. Minor *TMR* curves of a GdO_x-pMTJ measured at RT in the fresh state (black) and after 1200 s (red) and 2400 s (blue) at 200 ^{o}C with no applied voltage.

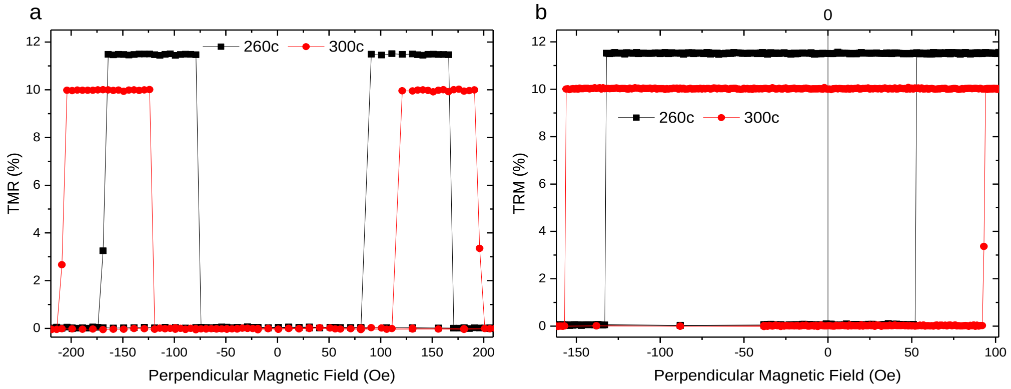

Supplementary Figure 8. Dependence of H_{IC} with PMA. (a) Full *TRM* loops of a pMTJ after annealing at 260 °C for 60 s, then subsequently at 300 °C for 180 s. **(b)** Corresponding minor loops showing decrease in $\mathbf{H}_{IC}$ despite increasing PMA contrary to the model proposed Moritz et al.[12] discussed in Supplementary Note 4.

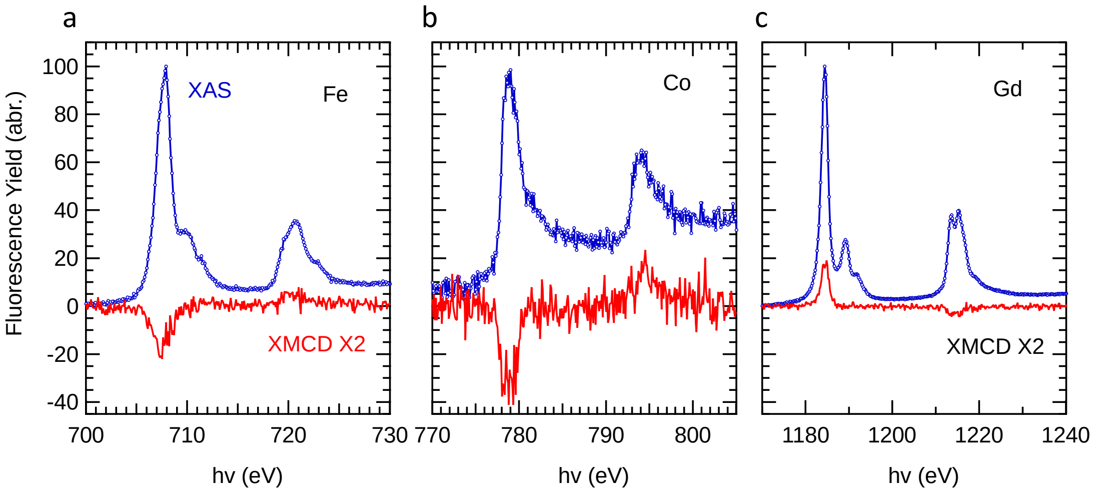

Supplementary Figure 9. Fluorescence yield XAS and XMCD spectra. Fluorescence yield XAS and XMCD spectra for Fe, Co and Gd in the pMTJ before voltage was applied. **(a)** Fe L edges **(b)** Co L edges **(c)** Gd M edges.

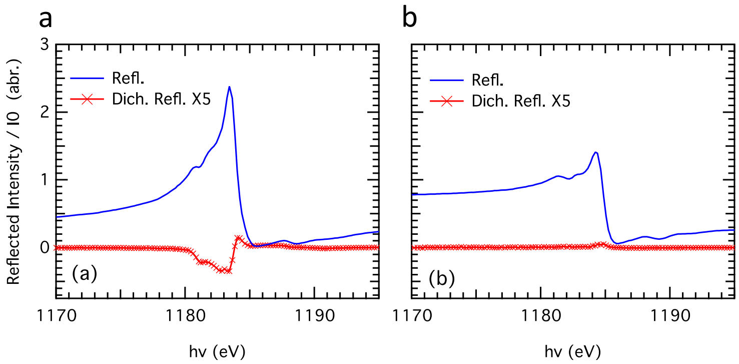

Supplementary Figure 10. Reflectivity yield XAS and XMCD spectra. Dichroic reflectivity as a function of photon energy at the Gd M_5 edge **(a)** On a CoFeB/GdO_x bilayer sample **(b)** On a GdO_x single layer sample.

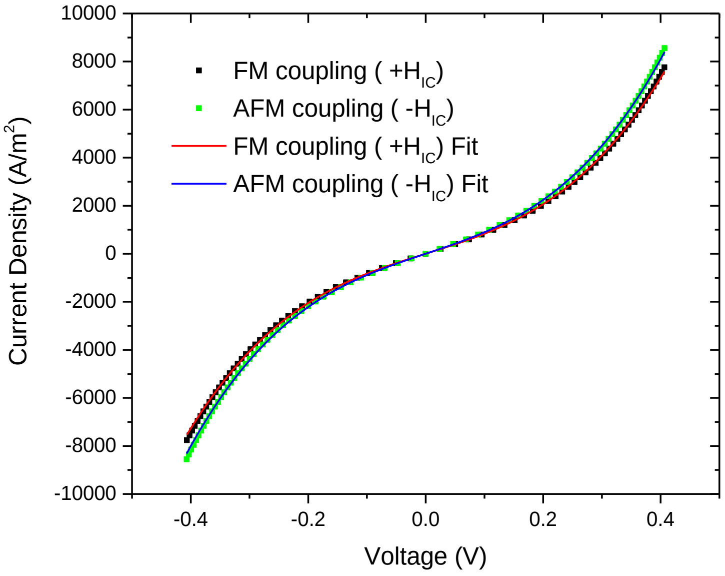

Supplementary Figure 11. IV curves. Representative *IV* curves of GdO_x–pMTJs in both the FM (black) and AFM (green) states as well as BDR fits for both states FM (red) and AFM (blue). If the observed Gd magnetic moment was in part due to unoxidized free Gd metal left in the tunnel barriers, the *IV* characteristics of the GdO_x-pMTJs at high bias voltage is expected to be substantially different from that of normal tunnel junctions. In such a case, a very low barrier height is expected due to defect-assisted tunneling. *IV* curves of the GdO_x-pMTJs, however, behave similarly to what is observed in MTJs with amorphous AlO_x. Fittings by the BDR model[2] yield d_{FM} =1.869 ± 0.005 nm, $\bar{\varphi}_{FM}$ = 1.157 ± 0.007 eV, $\Delta\varphi_{FM}$ = 0.016 ± 0.04 eV, d_{AFM} =1.879 ± 0.005 nm, $\bar{\varphi}_{AFM}$ = 1.127 ± 0.006 eV, $\Delta\varphi_{AFM}$ = -0.019 ± 0.04 eV in the FM and AFM states respectively. The height of about 1eV as shown for both states is comparable with that observed in normal AlO_x-MTJs[3]. The relatively large resistance of the FM state as shown in Fig. 4c is reflected by the larger barrier height for the FM state.

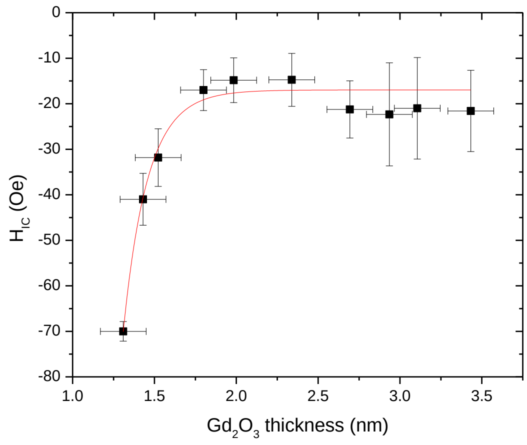

Supplementary Figure 12. H$_{IC}$ vs the thickness of the tunnel barrier. H$_{IC}$ was measured in pMTJs with the initial state (before application of V_{SET}).

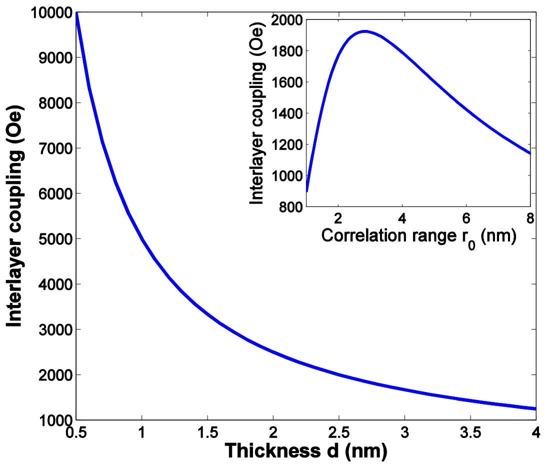

Supplementary Figure 13. Possible origin of the observed VCIC effect. The variation of **H**$_{IC}$ with distance between two layers with S = 3 and n = 1. Inset: the **H**$_{IC}$ vs correlation length.

Supplementary Note 1: Magnetic properties of continuous films

Supplementary Fig. 2a shows hysteresis loops of a continuous film measured by vibrating sample magnetometer (Microsense, EZ9 series) with magnetic fields applied in the in-plane and out-of-plane directions. The structure of the film is Si-wafer/SiO_2/Ta(8 nm)/Ru(10 nm) /Ta(7 nm)/$Co_{20}Fe_{60}B_{20}$(0.8 nm)/GdO_x(2.5 nm)/Ta(7 nm)/Ru(20 nm). The saturation magnetization and perpendicular anisotropy field are determined to be 800 emu/cc and 6.5 kOe, respectively. These values give rise to a PMA energy density of 2.6 x 10^6 erg/cc, which is comparable with CoFeB-MgO pMTJs[4,5]. In order to determine the interfacial perpendicular energy density, a series of samples with different CoFeB thickness were measured as shown in Supplementary Fig. 2b. The background signals from the substrate and sample holder have been subtracted.

To distinguish between the hard and soft layers of the pMTJ, we fabricated a pMTJ without the bottom CoFeB layer and compared it with a full pMTJ. Supplementary Fig. 3 shows hysteresis loops for the two different samples. The top CoFeB switches at smaller fields, demonstrating that the soft layer in the full MTJ is the top CoFeB while the hard layer is the bottom CoFeB. Samples again have GdO_x thickness of 2.5 nm as in Supplementary Fig. 2.

Supplementary Note 2: VCMA effect

In addition to the VCIC effect discussed in this paper this system is the first other than CoFeB/MgO to display the voltage controlled magnetic anisotropy (VCMA) effect. The VCMA effect is a volatile effect where by the *TMR* curve can be temporarily modified during the application of high bias voltage. As shown in Supplementary Fig. 4 the application of high positive bias increases the coercivity of the hard magnetic layer and decreases the coercivity of the soft

magnetic layer, thus broadening the antiparallel plateau. Conversely a high negative bias decreases the coercivity of the hard layer and increases the coercivity of the soft layer, narrowing the antiparallel plateau. In addition high bias of either polarity decreases dramatically the resistance and *TMR* of the junction.

Supplementary Note 3: Control experiment with MgO-pMTJ and annealing experiment without V_{SET}

To confirm that the change in interlayer coupling was a unique property due to the GdO_x, a comparison experiment was run with an MgO-pMTJ, under the same testing conditions as the GdO_x-pMTJ described in Fig. 3. No change was seen in the $\mathbf{H}_{IC}$ of the MgO-pMTJ as shown in Supplementary Fig. 6. The MgO thickness for this sample is 2 nm with a corresponding electric field of $\mathbf{E}_{\mathrm{MgO}}$ = 0.3 V/nm, which is larger than $\mathbf{E}_{\mathrm{GdOx}}$ = 0.17 V/nm.

To further verify that the change in interlayer coupling in GdO_x-pMTJs was due to the applied voltage, instead of any annealing effects at 150-200 ^{o}C, we tested the $\mathbf{H}_{IC}$ of a GdO_x-pMTJ after annealing with no voltage applied. Supplementary Fig. 7 shows that even with heating for far longer than that used in the VCIC experiments, there is no change in $\mathbf{H}_{IC}$, demonstrating that the change of $\mathbf{H}_{IC}$ is indeed due to the voltage applied to the pMTJs.

Supplementary Note 4: Dependence of $\mathbf{H}_{\mathrm{IC}}$ with PMA of GdO_x-pMTJ

According to a model proposed by Moritz et al.[6], the interlayer coupling in (Co/Pt)-Ru-(Co/Pt) PMA spinvalves can be explained by an extended Neel-type orange peel coupling induced by correlated roughness. In this model, the AFM coupling is stronger in samples with larger PMA, which cannot explain the decrease of AFM coupling illustrated in Supplementary Fig. 8. A pMTJ was measured after annealing at 260 °C for 60 s, then subsequently after

annealing at 300 °C for 180 s. The PMA of both of the magnetic layers was higher after the 300 °C annealing, as is evident from larger switching fields for both the hard and soft CoFeB layers as shown in Supplementary Fig. 8a. This increase in PMA, however, corresponds to a decrease in AFM coupling as shown in Supplementary Fig. 8b, with $\mathbf{H}_{IC}$ dropping from -40 Oe to -31.5 Oe after 300 °C annealing. This suggests that the AFM coupling in the CoFeB/GdO_x/ CoFeB system cannot be explained by the Moritz model.

Supplementary Note 5: In situ *TMR* and X-ray absorption and X-ray magnetic circular dichroism

Soft x-ray absorption (XAS) and magnetic circular dichroism (XMCD) spectra were collected at beamline 4-ID-C of the Advanced Photon Source. We have designed a special pattern for the pMTJ so both *TMR* and XMCD signals can be obtained simultaneously while sweeping the magnetic field. A large junction size of D = 200 μm and a thicker GdO_x barrier thickness (>3 nm) were used for this purpose. The RT data on an as-prepared junction are shown in Supplementary Fig. 9. Data were collected at the Fe and Co $L_{2,3}$ edges and the Gd $M_{4,5}$ edges, with an incident beam angle of 70° with respect to the film plane and with an applied magnetic field of 3.5 kOe normal to the film plane. The high incident angle was purposely used to improve the sensitivity to the buried magnetic layers of interest[7]. The XMCD (XAS) spectra are given by the difference (average) of the x-ray absorption spectra of the right and left circularly polarized x-rays detected by fluorescence yield. The data were normalized by the x-ray intensity and then by the edge jump before and after the L_3 edge of the average absorption spectra. On the high energy side of the Fe L_3 main peak, a clear shoulder has been found, indicating the existence of a high oxidation state of Fe, either Fe_3O_4 or α-Fe_2O_3[8,9]. The spin and orbital components of the Co, Fe and Gd magnetization were estimated using the XMCD sum-rules[10]. The explicit forms at $L_{2,}$

$_3$ edges of the 3d transition metals in unit of μ_B/ion are:

$$m_{\text{orb}} = -\frac{4}{3}\, n_h \frac{\int_{\text{L3+L2}}(\mu_+ - \mu_-)dE}{\int_{L3+L2}(\mu_+ + \mu_-)dE} \quad (1)$$

$$m_{\text{spin}} = -n_h \left(1 + \frac{7<T_z>}{2<S_z>}\right)^{-1} \frac{\int_{L3}(\mu_+ - \mu_-)dE - 2\int_{L2}(\mu_+ - \mu_-)dE}{\int_{\text{L3+L2}}(\mu_+ + \mu_-)dE}. \quad (2)$$

The explicit forms at $M_{4,5}$ edges for 4f rare earth metals are:

$$m_{\text{orb}} = -3n_h \frac{\int_{\text{M5+M4}}(\mu_+ - \mu_-)dE}{\int_{\text{M5+M4}}(\mu_+ + \mu_- + \mu_0)dE} \quad (3)$$

$$m_{\text{spin}} = -\frac{3}{2} n_h \left(1 + 3\frac{<T_z>}{<S_z>}\right)^{-1} \frac{2\int_{\text{M5}}(\mu_+ - \mu_-)dE - 3\int_{\text{M4}}(\mu_+ - \mu_-)dE}{\int_{\text{M5+M4}}(\mu_+ + \mu_- + \mu_0)dE}, \quad (4)$$

where μ_+ (μ_-) is the absorption intensity with left (right) circularly polarized x-rays; μ_0 was approximated by the average value of μ_+ and μ_- during the calculation; n_h is the number of holes in the 3d (4f) shells; $<Tz>$ and $<Sz>$ are the expectation value of the spin operator and the magnetic dipole operator, respectively. The factor $\frac{<T_z>}{<S_z>}$ was assumed to be small and ignored. The photon incident angle (70°), and the circular polarization of 96% have been accounted for in the calculations. During the calculations, the hole numbers $n_h^{3d} = 2.5$ for Co, $n_h^{3d} = 4.2$ for Fe (in FeO_x), and $n_h^{4f} = 7$ for Gd were used. Clearly the magnetic moment from Gd ions are antiparallel to that of Fe and Co, as shown in Supplementary Fig. 9. At RT, the spin (orbital) moment was calculated to be 0.51 (0.07) μ_B per Fe atom, 1.26 (0.09) μ_B per Co atom, and -0.46 (-0.20) μ_B per Gd atom, respectively. The accuracy of the sum rule becomes worse as the number of electrons decreases in the 3*d* orbital[11]. It could significantly underestimate the Fe spin moment by ~ 30%; on the other hand, the accuracy of the spin moments of Co and Gd are about 10%. Due the much larger Fe concentration in the $Co_{20}Fe_{60}B_{20}$ electrodes, the magnetic field dependence of XMCD signal was only performed for Fe.

In order to further confirm that the large Gd magnetization is due to the proximity effect, we have measured the dichroic reflectivity of two unpatterned reference samples: a CoFeB/GdO_x bilayer and a GdO_x single layer. The resonant magentic scattering data has much better signal/noise ratio making it very sensitive to the weak magnetic signals from buried interfaces[12,13].Experiments were conducted with an incident angle of 10 deg. with ±5 kOe in-plane fields. The data shown in Supplementary Fig. 10 were normalized by the direct beam intensity. There is a strong magnetic scattering near the Gd M_5 edge in the CoFeB/GdO_x bilayer, but not in the single GdO_x layer sample, indicating that the strong Gd magnetic signal is indeed due to the proxmity effect.

Supplementary Note 6: Possible origin of the observed VCIC effect

Previous theories[14–17] of voltage controlled interlayer coupling did not consider the direct manipulation of $\mathbf{H}_A$ and M_S or, more significantly, a large induced magnetic moment in the barrier. Therefore, these theories cannot be applied to the observed effects in the present GdO_x pMTJs. The average ferromagnetic moment of Gd ions induced by CoFeB is 0.6 μ_B per Gd^{3+} ion, which is nearly 20 times larger than the induced moment of Pt in Pt/Fe bilayers[18]. Interface-induced magnetization in tunnel barriers has also been observed in other MTJ systems, where it affects the spin-dependent transport[19]. We do not expect, however, the induced moment of Gd^{3+} to significantly affect the transport properties of our junctions due to the localized nature of *f* electrons. It may, however, play an important role in the magnetic properties, e.g., the coupling of the two CoFeB layers in the pMTJ. Generally in the magnetic proximity effect, the depth distribution of the induced moments in the NM layers varies from system to system. For example, it is estimated that 90% of the induced moments in Pt on Co exist only in the first four monolayers from the interface, with a characteristic decay length of 0.41 nm[20]. In other cases,

however, the induced magnetization can extend much deeper into the NM layer, such as in Bi_2Se_3/EuS where the first 2 nm of Bi_2Se_3 was found to be ferromagnetic[21].

The thickness dependence of the $\mathbf{H}_{IC}$ in the initial state of the GdO_x-pMTJ is plotted in Supplementary Fig. 12. The AFM coupling in our samples is extended to much thicker barriers compared to MBE grown Fe/MgO/Fe MTJs with in-plane magnetic anisotropy[22,23]. The overall shape of the curve, however, resembles that of Fe/MgO/Fe MTJs with a sharp increase of $\mathbf{H}_{IC}$ when the thickness of GdO_x is reduced below 1.5 nm. This barrier thickness dependence of $\mathbf{H}_{IC}$ is similar to that of sputtered CoFeB/MgO multilayers, where the AFM coupling persists to d_{MgO} > 2.2 nm[24] with a very interesting observation of multilevel switching, and of $(Pt/Co)_3$-NiO-$(Pt/Co)_3$ multilayers, where coupling continues to d_{NiO} > 2 nm[25]. Due to the induced moment of Gd ions in the barrier, the nonmagnetic portion of the GdO_x barrier (corresponding to the thickness of MgO in previous studies) is thinner than the total GdO_x thickness, which may partly explain why the AFM coupling here is observed with a thicker barrier in the GdO_x-pMTJs. A Neutron diffraction experiment is planned to probe the distribution of induced Gd moments. Under present conditions, V_{SET} (0.5-0.8 V) needs to be applied to the GdO_x-pMTJs for between a few tens of seconds and a few minutes, which could lead to the breakdown of the tunnel barrier when the GdO_x is less than 2 nm thick. Due to this limitation, most VCIC experiments in this work were performed on pMTJs with GdO_x thicker than 2 nm. This situation in principle can be improved once GdO_x with higher quality can be fabricated. Therefore we expect to observe the same VCIC in junctions with thin barriers (GdO_x < 1.5 nm) when the quality of the GdO_x barrier is improved.

In the following, we describe the VCIC with thick GdO_x (> 2 nm) with a model considering the voltage-driven oxidation level changes of Fe, the large induced moment of the

Gd ions that is proportional to the amount of free Fe, and a voltage dependent distribution of correlated moments in the Gd ions. Since we have shown that oxygen in the GdO_x barrier can be reversibly moved toward or away from the interface by applying voltage, we propose that the correlation of the magnetic moment distributions between the two interfaces created by the oxygen transportation may be responsible for the observed VCIC. The XMCD has shown that the Gd ions display significant magnetic moments due to the proximity effect with CoFeB. These large induced Gd moments may contribute to the interplay coupling via dipolar interaction, especially in samples with thick barriers (> 2 nm).

Consider two thin magnetic layers separated by a distance d. The coupling energy between these two layers can be expressed as:

$$E = \frac{\mu_0}{4\pi} \int d\boldsymbol{\rho}_1 d\boldsymbol{\rho}_2 \frac{\boldsymbol{m}_1(\boldsymbol{\rho}_1)\cdot\boldsymbol{m}_2(\boldsymbol{\rho}_2) - 3[\boldsymbol{m}_1(\boldsymbol{\rho}_1)\cdot\hat{\boldsymbol{r}}_{12}][\boldsymbol{m}_2(\boldsymbol{\rho}_2)\cdot\hat{\boldsymbol{r}}_{12}]}{r_{12}^3} \tag{5}$$

where $m_1(m_2)$ is the magnetic moment at the position $\rho_1(\rho_2)$, $r_{12} = \rho_1 - \rho_2 + d\hat{z}$, $r_{12} = |r_{12}|$, and $\hat{r}_{12} = r_{12}/r_{12}$. For perpendicularly magnetized layers, the above integration is identically zero if the magnetic moments in each layer are uniformly distributed. In the present case, the migration of the oxygen vacancies to the two interfaces may create a highly non-uniform distribution of the interface moments. Let's introduce an impurity moment $\delta m_i(\rho_i) = m_i(\rho_i) - \overline{m}_i$, where $\overline{m}_i$ is the optimally and uniformly magnetized magnetic moment of the i-th layer. One may think of m_i as a Gd ion that is polarized by a Fe atom next to it. Consequently m_i is zero if the neighboring Fe atom is oxidized by O^{2-} driven by the applied voltage. To estimate the magnitude of the coupling from such non-uniform distributions, we define the correlation function between the distributions of the magnetic moments of the two layers,

$$\xi_{12}(\boldsymbol{\rho}_1 - \boldsymbol{\rho}_2) = < \delta\boldsymbol{m}_1(\boldsymbol{\rho}_1) \cdot \delta\boldsymbol{m}_2(\boldsymbol{\rho}_2) > - < \delta\boldsymbol{m}_1(\boldsymbol{\rho}_1) > \cdot < \delta\boldsymbol{m}_2(\boldsymbol{\rho}_2) > \tag{6}$$

where <> refers to the spatial average over the plane of the layers (for a fixed $\rho_1 - \rho_2$). Due to the much stronger PMA of the bottom CoFeB layer (Supplementary Figure 3), the change in magnetic properties of top CoFeB layer by voltage to be much larger than that of bottom CoFeB, which is supported by Fig. 4b. We assume that the applied voltage from the positive to negative polarity results in a change of the correlation function from the correlated ($\xi_{12} > 0$) to the anti-correlated ($\xi_{12} < 0$) state, i.e., the oxidation level (therefore the induced moment from Gd ions) of the two interfaces is likely similar if $|\rho_1 - \rho_2| < r_0$ for a positive voltage, where r_0 is the correlation length. For a negative voltage, the oxidation level of the two interfaces is likely to be less similar, giving rise to $\xi_{12} < 0$. With the above assumptions, we can now estimate the coupling field $\boldsymbol{H}_{IC} \equiv -\,\partial E/\partial\delta\boldsymbol{m}_1$. The z-component of the coupling field is thus,

$$\boldsymbol{H}_{IC}^{z} = \frac{\mu_0}{4\pi}\frac{\mu_B}{a^2}\int d^2\boldsymbol{\rho}\,\frac{2z^2-\rho^2}{(\rho^2+d^2)^{\frac{5}{2}}}\xi_{12}(\boldsymbol{\rho}) \tag{7}$$

If we take a simple correlation function $\xi_{12}(\boldsymbol{\rho}) = n^2S^2\theta(\boldsymbol{r}_0 - \boldsymbol{\rho})$ where n is the density of impurities, S is the spin, and θ is the step function, one can analytically integrate the above equation. In the inset of Supplementary Fig. 13, we show the coupling field as a function of the correlation range r_0. Taking the maximum value for the optimal r_0 for each thickness of the barrier, we show in Supplementary Fig. 13 the coupling field as a function of the barrier thickness with $n = 1$ and $S = 3$.

The GdO_x barrier in the pMTJ used in the XMCD experiment has a thickness of 3.4 nm, with an average induced moment of 0.6 μ_B per Gd ion as shown in Supplementary Note 5. If most of these moments reside in only the first monolayers of GdO_x (0.27 nm) next to CoFeB on both sides of the barrier, the induced moment per Gd ion can be as large as ~3.7 μ_B, which is the basis for using S = 3 in Equation 7. Alternatively, if we assume that the induced moment of the Gd ions resides in the first two monolayers (0.54 nm) next to CoFeB on both sides of the barrier, we have an average induced moment of ~1.8 μ_B per Gd ion, which can be described approximately by S = 2. For $n = 1$, S = 3, and $d = 2$ nm, we find H_{IC} to be about 2500 Oe as shown in Supplementary Fig. 13. With a more realistic approximation of n = 0.2 that corresponds to a 20% impurity density, we have $\mathbf{H}_{IC} \approx 100$ Oe with the sign determined by ξ_{12}, which is comparable the experimental results.

In conclusion, while the simple model based on the voltage driven correlation of the magnetic moments of two magnetic layers can roughly account for the sign change and the magnitude of $\mathbf{H}_{IC}$, there are parameters that are not fully justified. In particular, the detailed correlation function is unknown at the present time. Further experiments are needed to establish definitive mechanisms for the unique VCIC observed in GdO_x-pMTJs.

References

1. Wang, W. G., Jordan-Sweet, J., Miao, G. X., Ni, C., Rumaiz, A., Shah, L., Fan, X., Parson, P., Stearrett, R., Nowak, R., Moodera, J. S. & Xiao, J. Q. In situ characterization of rapid crystallization of amorphous CoFeB electrodes in CoFeB/MgO/CoFeB junctions during thermal annealing. *Appl. Phys. Lett.* **95,** 242501 (2009).
2. Brinkman, W. F., Dynes, R. C. & Rowell, J. M. Tunneling conductance of asymmetrical barriers. *J. Appl. Phys.* **41,** 1915–1921 (1970).
3. Landry, G. INTERFACIAL EFFECTS IN MAGNETIC TUNNELING JUNCTIONS. *Ph.D. Thesis* (2001).
4. Ikeda, S., Miura, K., Yamamoto, H., Mizunuma, K., Gan, H. D., Endo, M., Kanai, S., Hayakawa, J., Matsukura, F. & Ohno, H. A perpendicular-anisotropy CoFeB-MgO magnetic tunnel junction. *Nat. Mater.* **9,** 721–4 (2010).
5. Almasi, H., Hickey, D. R., Xu, M., Rosales, M. R., Nahar, S., Held, J. T., Mkhoyan, K. a & Wang, W. G. Enhanced tunneling magnetoresistance and perpendicular magnetic anisotropy in Mo / CoFeB / MgO magnetic tunnel junctions. *Appl. Phys. Lett.* **106,** 182406 (2015).
6. Moritz, J., Garcia, F., Toussaint, J. C., Dieny, B. & Nozières, J. P. Orange peel coupling in multilayers with perpendicular magnetic anisotropy: Application to (Co/Pt)-based exchange-biased spin-valves. *Europhys. Lett.* **65,** 123–129 (2007).
7. Liu, Y. & Ke, X. Interfacial magnetism in complex oxide heterostructures probed by neutrons and x-rays. *J. Phys. Condens. Matter* **27,** 373003 (2015).
8. Crocombette, J., Pollak, M., Jollet, F., Thromat, N. & Gautier-Soyer, M. X-ray-absorption spectroscopy at the Fe L_ {2, 3} threshold in iron oxides. *Phys. Rev. B* **52,** 3143–3150 (1995).
9. Regan, T., Ohldag, H., Stamm, C., Nolting, F., Lüning, J., Stöhr, J. & White, R. Chemical effects at metal/oxide interfaces studied by x-ray-absorption spectroscopy. *Phys. Rev. B* **64,** 1–11 (2001).
10. Carra, P., Thole, B. T., Altarelli, M. & Wang, X. X-ray circular dichroism and local magnetic fields. *Phys. Rev. Lett.* **70,** 694–697 (1993).
11. Piamonteze, C., Miedema, P. & De Groot, F. M. F. Accuracy of the spin sum rule in XMCD for the transition-metal L edges from manganese to copper. *Phys. Rev. B - Condens. Matter Mater. Phys.* **80,** 1–12 (2009).

12. Liu, Y., Tornos, J., te Velthuis, S. G. E., Freeland, J. W., Zhou, H., Steadman, P., Bencok, P., Leon, C. & Santamaria, J. Induced Ti magnetization at La0.7Sr0.3MnO3 and BaTiO3 interfaces. *APL Mater.* **4,** 46105 (2016).

13. KAO, C. C., Chen, C. T., Jhonson, E. D., Hastings, J. B., J., H. L., Ho, G. H., G. Meigs, M. J. Brot, Hulbert, S. L., Idzerda, Y. U. & Vettier, C. dichroic interference effects in circularly polarized sorf x ray resonant magnetic scattering. *Phys. Rev. B* **50,** 9599–9602 (1994).

14. You, C. & Bader, S. Prediction of switching/rotation of the magnetization direction with applied voltage in a controllable interlayer exchange coupled system. *J. Magn. Magn. Mater.* **195,** 488–500 (1999).

15. You, C. Y. & Suzuki, Y. Tunable interlayer exchange coupling energy by modification of Schottky barrier potentials. *J. Magn. Magn. Mater.* **293,** 774–781 (2005).

16. Zhuravlev, M. Y., Vedyayev, a V & Tsymbal, E. Y. Interlayer exchange coupling across a ferroelectric barrier. *J. Phys. Condens. Matter* **22,** 352203 (2010).

17. Fechner, M., Zahn, P., Ostanin, S., Bibes, M. & Mertig, I. Switching magnetization by 180 degree with an electric field. *Phys. Rev. Lett.* **108,** 1–5 (2012).

18. Geprägs, S., Meyer, S., Altmannshofer, S., Opel, M., Wilhelm, F., Rogalev, A., Gross, R. & Goennenwein, S. T. B. Investigation of induced Pt magnetic polarization in Pt/Y 3Fe5O12 bilayers. *Appl. Phys. Lett.* **101,** (2012).

19. Liu, Y., Cuellar, F. A., Sefrioui, Z., Freeland, J. W., Fitzsimmons, M. R., Leon, C., Santamaria, J. & Te Velthuis, S. G. E. Emergent spin filter at the interface between ferromagnetic and insulating layered oxides. *Phys. Rev. Lett.* **111,** 1–5 (2013).

20. Suzuki, M., Muraoka, H., Inaba, Y., Miyagawa, H., Kawamura, N., Shimatsu, T., Maruyama, H., Ishimatsu, N., Isohama, Y. & Sonobe, Y. Depth profile of spin and orbital magnetic moments in a subnanometer Pt film on Co. *Phys. Rev. B - Condens. Matter Mater. Phys.* **72,** 1–8 (2005).

21. Katmis, F., Lauter, V., Nogueira, F. S., Assaf, B. A., Jamer, M. E., Wei, P., Satpati, B., Freeland, J. W., Eremin, I., Heiman, D., Jarillo-Herrero, P. & Moodera, J. S. A high-temperature ferromagnetic topological insulating phase by proximity coupling. *Nature* 1–11 (2016). doi:10.1038/nature17635

22. Faure-Vincent, J., Tiusan, C., Bellouard, C., Popova, E., Hehn, M., Montaigne, F. & Schuhl, a. Interlayer Magnetic Coupling Interactions of Two Ferromagnetic Layers by Spin Polarized Tunneling. *Phys. Rev. Lett.*

89, 107206 (2002).

23. Katayama, T., Yuasa, S., Velev, J., Zhuravlev, M. Y., Jaswal, S. S. & Tsymbal, E. Y. Interlayer exchange coupling in Fe/MgO/Fe magnetic tunnel junctions. *Appl. Phys. Lett.* **89,** 112503 (2006).

24. Moubah, R., Magnus, F., Warnatz, T., Palsson, G. K., Kapaklis, V., Ukleev, V., Devishvili, A., Palisaitis, J., Persson, P. O. Å. & Hjörvarsson, B. Discrete Layer-by-Layer Magnetic Switching Fe/MgO Superlattices. *Phys. Rev. Appl.* **5,** 44011 (2016).

25. Liu, Z. Y. & Adenwalla, S. Oscillatory interlayer exchange coupling and its temperature dependence in [Pt/Co]3/NiO/[Co/Pt]3 multilayers with perpendicular anisotropy. *Phys. Rev. Lett.* **91,** 37207 (2003).
